# Frequency of peripheral diseases in Korean patients with ankylosing spondylitis and the effectiveness of adalimumab

**DOI:** 10.1111/1756-185X.13917

**Published:** 2020-07-29

**Authors:** Sang‐Hoon Lee, Won Park, Sung Won Lee, Hyun Ah Kim, Jung‐Yoon Choe, Sang‐Heon Lee, Shin‐Seok Lee, Sung‐Hwan Park, Min‐Chan Park, Dong‐Hyuk Sheen, Hye Soon Lee, Yeon‐Ah Lee, Yusun Lee, Tae‐Hwan Kim

**Affiliations:** ^1^ Department of Rheumatology Hospital at Gang dong College of Medicine Kyung Hee University Seoul Korea; ^2^ Division of Rheumatology Inha University College of Medicine Incheon Korea; ^3^ Division of Rheumatology Dong‐A University College of Medicine Busan Korea; ^4^ Division of Rheumatology Hallym University Sacred Heart Hospital Hallym University School of Medicine Anyang Korea; ^5^ Division of Rheumatology Catholic University of Daegu School of Medicine Daegu Korea; ^6^ Division of Rheumatology Konkuk University Medical Center Seoul Korea; ^7^ Division of Rheumatology Chonnam National University Hospital Gwangju Korea; ^8^ Division of Rheumatology College of Medicine Seoul St. Mary's Hospital The Catholic University of Korea Seoul Korea; ^9^ Division of Rheumatology Department of Internal Medicine Yonsei University College of Medicine Seoul Korea; ^10^ Division of Rheumatology Eulji University Hospital Daejeon Korea; ^11^ Division of Rheumatology Hanyang University Guri Hospital Guri Korea; ^12^ Division of Rheumatology Kyung Hee University Hospital Seoul Korea; ^13^ AbbVie Ltd Seoul Korea; ^14^ Hanyang University Hospital for Rheumatic Diseases Seoul Korea

**Keywords:** adalimumab, ankylosing spondylitis, dactylitis, enthesitis, Korea, peripheral arthritis, peripheral disease

## Abstract

**Aim:**

Peripheral features contribute to disease burden in ankylosing spondylitis (AS). This study investigated the frequency of peripheral disease and effectiveness of adalimumab among Korean patients with AS.

**Methods:**

Peripheral disease was evaluated in consecutively enrolled patients with active AS (Bath Ankylosing Spondylitis Disease Activity Index [BASDAI] score ≥ 4). An adult subpopulation was subsequently enrolled in a prospective, observational study and received adalimumab 40 mg, every 2 weeks. During a 52‐week follow‐up, AS disease activity was assessed by BASDAI score, and effectiveness in peripheral disease assessed via changes in Maastricht Ankylosing Spondylitis Enthesitis Score (MASES; 0‐13), swollen joint and tender joint counts (SJC, 0‐44; TJC, 0‐46), and dactylitic digits from baseline.

**Results:**

Of 1161 Korean patients with AS, 178 (15.3%) and 306 (26.4%) had enthesitis and peripheral arthritis, respectively; dactylitis was diagnosed in 28 patients (2.4%). Of 201 patients enrolled in the observational study, 46.3%, 33.3%, and 3.0% had enthesitis, peripheral arthritis, and dactylitis, respectively. Overall, 75.1% of patients achieved >50% improvement in BASDAI score by week 12. Mean MASES was significantly reduced from 2.67 at baseline to 0.85 and 0.34 at weeks 12 and 52, respectively (*P* < .0001). Similarly, SJC and TJC improved significantly from 2.58 and 3.49 at baseline to 0.80 and 1.68, respectively, by week 12 (*P* < .0001). Dactylitis was resolved in all affected patients by week 28.

**Conclusion:**

Of these Korean patients with AS, those who received adalimumab demonstrated higher prevalence for peripheral symptoms and, subsequently, adalimumab treatment improved peripheral features of their AS.

## 1 INTRODUCTION

Ankylosing spondylitis (AS) is a chronic rheumatic disorder, primarily associated with inflammation of the spine.^1^ In addition, peripheral disease, encompassing enthesitis, dactylitis and peripheral arthritis, contributes to the overall disease burden of this condition.^2^ In Korea an increasing prevalence of AS is being observed, with 47%–70% of patients reported to experience associated peripheral disease.^3^, ^4^, ^5^

Alongside nonpharmacological interventions, nonsteroidal anti‐inflammatory drugs (NSAIDs) represent first‐line therapy for AS.^1^ In patients who require additional treatment, tumor necrosis factor‐alpha (TNFα) inhibitors are well tolerated and have been shown to improve clinical symptoms.^6^ Adalimumab (anti‐TNFα) has demonstrated efficacy in the reduction of symptoms in AS in multiple randomized controlled trials (RCTs), and is approved for the treatment of AS in Korea.^7^, ^8^, ^9^ More recently, the sustained benefits of adalimumab treatment on spinal mobility, health‐related quality of life, physical function and disease activity have been confirmed in a long‐term study over a period of 5 years.^10^

A meta‐analysis of 8 studies in AS estimated the prevalence of arthritis, enthesitis, and dactylitis as 29.7%, 28.8% and 6.0%, respectively,^11^ although individual studies have reported a history of peripheral disease in more than 50% of patients.^12^ In a multicenter study of patients with AS in Europe, frequency of enthesitis and peripheral arthritis were 54.9% and 22.5%, respectively.^13^ Efficacy of adalimumab treatment on peripheral disease features in AS has previously been shown. In an RCT of over 300 patients with active AS, adalimumab significantly reduced enthesitis versus placebo over 24 weeks.^8^ Consistent with these data, in an uncontrolled, open‐label study in 1250 patients, adalimumab therapy was shown to be effective in the reduction of peripheral arthritis, and associated with a significant improvement in enthesitis by week 12.^13^

In Korea, studies investigating the clinical spectrum of AS are limited; to date, no studies have examined the efficacy of adalimumab on peripheral joint involvement in AS. Enthesitis has a reported prevalence of 53%,^4^ while 2 separate studies conducted in Korea estimated peripheral arthritis symptoms in 47% and 70% of AS patients.^3^, ^4^ The occurrence of peripheral arthritis has also been significantly associated with a higher risk of TNFα inhibitor treatment discontinuation in Korean patients with AS.^14^ Thus, we conducted an observational, prospective study to investigate the frequency of peripheral disease in Korean patients with AS, and to determine the efficacy of adalimumab treatment on these extra‐axial manifestations.

## 2 PATIENTS AND METHODS

### 2.1 Study population

Patients with AS were enrolled consecutively from 13 sites in South Korea (Table S1) to determine the frequency of peripheral disease. Patients eligible for the adalimumab treatment study were either adalimumab‐naïve or could be switched from other TNFα inhibitor therapies. Patients who had received prior treatment with adalimumab required a minimum of 70 days without adalimumab prior to first administration of study medication. Inclusion criteria were adults aged ≥ 19 years who had a diagnosis of AS for ≥3 months (according to the 1984 modified New York criteria), and eligible for adalimumab treatment according to routine rheumatological practice. Patients were required to have active AS, as defined by a Bath Ankylosing Spondylitis Disease Activity Index (BASDAI) score ≥4, despite previous treatment with ≥2 NSAIDs for >3 months as per the Korean AS reimbursement guidelines. Exclusion criteria included pregnant or breastfeeding female patients contraindicated to any TNFα inhibitors, and individuals participating in other clinical trials.

### 2.2 Study design

We conducted a prospective, noninterventional, observational study between December 2014 and August 2017 (NCT02333383). Frequency of peripheral disease was assessed in an initial cohort of patients with AS, from which a subpopulation of adult subjects was enrolled according to the criteria described. Patients were treated with subcutaneous administrations of adalimumab 40 mg once every 2 weeks, at the discretion of the treating physician and in accordance with clinical practice and the drug label. The study was approved by the institutional review boards of each university or hospital and was conducted in accordance with the Declaration of Helsinki and Guidelines for Good Clinical Practice. Written informed consent was obtained from all participants.

Patient baseline data, including demographics and disease characteristics, were assessed prior to first dose of adalimumab (week 0). Presence of peripheral disease, including peripheral arthritis, enthesitis and dactylitis, was also assessed. Follow‐up study visits were conducted 12, 28, 36 and 52 weeks thereafter, during which BASDAI scores were measured to assess AS disease activity. Peripheral disease responses were captured via changes in the Maastricht Ankylosing Spondylitis Enthesitis Score (MASES; 0‐13), alongside assessments for plantar fascia, tender joint counts (TJC; 0‐46) and swollen joint counts (SJC; 0‐44), and for dactylitis counts (0‐20). Enthesitis was defined as the presence of at least 1 inflamed enthesis per MASES, or enthesitis of the plantar fascia of the foot. Peripheral arthritis was defined as at least 1 swollen joint, excluding the hip joints, while dactylitis was measured by a simple count of dactylitic digits. All patients received a follow‐up assessment within approximately 70 days of their last dose of adalimumab during the study period. Adverse events (AEs) were recorded via case report forms, and classified by system organ class and preferred terms as per the *Medical Dictionary for Regulatory Activities* hierarchy.

### 2.3 Outcomes

The study's primary endpoint was the frequency of peripheral disease (peripheral arthritis, enthesitis and dactylitis) at baseline. Secondary endpoints were assessed at each study visit (12, 28, 36 and 52 weeks) and included: the percentage of patients with a 50% improvement in BASDAI score (BASDAI 50) from baseline; change in MASES from baseline; prevalence of enthesitis of the plantar fascia; change of TJC and SJC in patients with peripheral arthritis, defined as ≥1 swollen joint at baseline; and dactylitis score from baseline.

### 2.4 Statistical analysis

Descriptive statistics were used to present patient demographics, disease and medication data. Continuous variables are described using mean and standard deviation (SD) values, as well as median and range (minimum–maximum) measurements. Categorical variables are presented using frequency and proportions. All missing data were treated as missing values in the analysis. The study sample size was based on the frequency of peripheral disease in Korean patients^3^, ^4^; using a 95% confidence interval (CI) and 15% CI width, sample size was estimated at 144 patients with peripheral arthritis, and 171 patients with enthesitis. A final sample size of 200 was determined for enrollment taking into account a 20% dropout rate.

The study population comprised intention‐to‐treat (ITT) and per protocol (PP) sets. The ITT set included patients who received at least 1 dose of adalimumab and had peripheral disease assessed at baseline, while the PP subset of patients completed the study without deviation and met inclusion and exclusion criteria.

Post hoc analyses of secondary outcomes were performed for patient subgroups according to total number of adalimumab injections and use of prior TNFα inhibitor treatment. Analyses were performed using Fisher's exact test, and Chi‐square test.

All statistical analyses were performed using SAS software version 9.4 (SAS Institute Inc, Cary, NC, USA). All statistical tests were 2‐tailed paired *t* tests, and *P* < .05 was considered statistically significant using Fisher's exact test, nonparametric Wilcoxon signed rank test or signed rank test.

## 3 RESULTS

### 3.1 Patient clinical characteristics

In total, 1161 patients with AS were evaluated for study inclusion and for frequency of peripheral disease; mean (SD) age was 40.5 (12.1) years, and 81.1% (941) of the patients were male.

**FIGURE 1:**
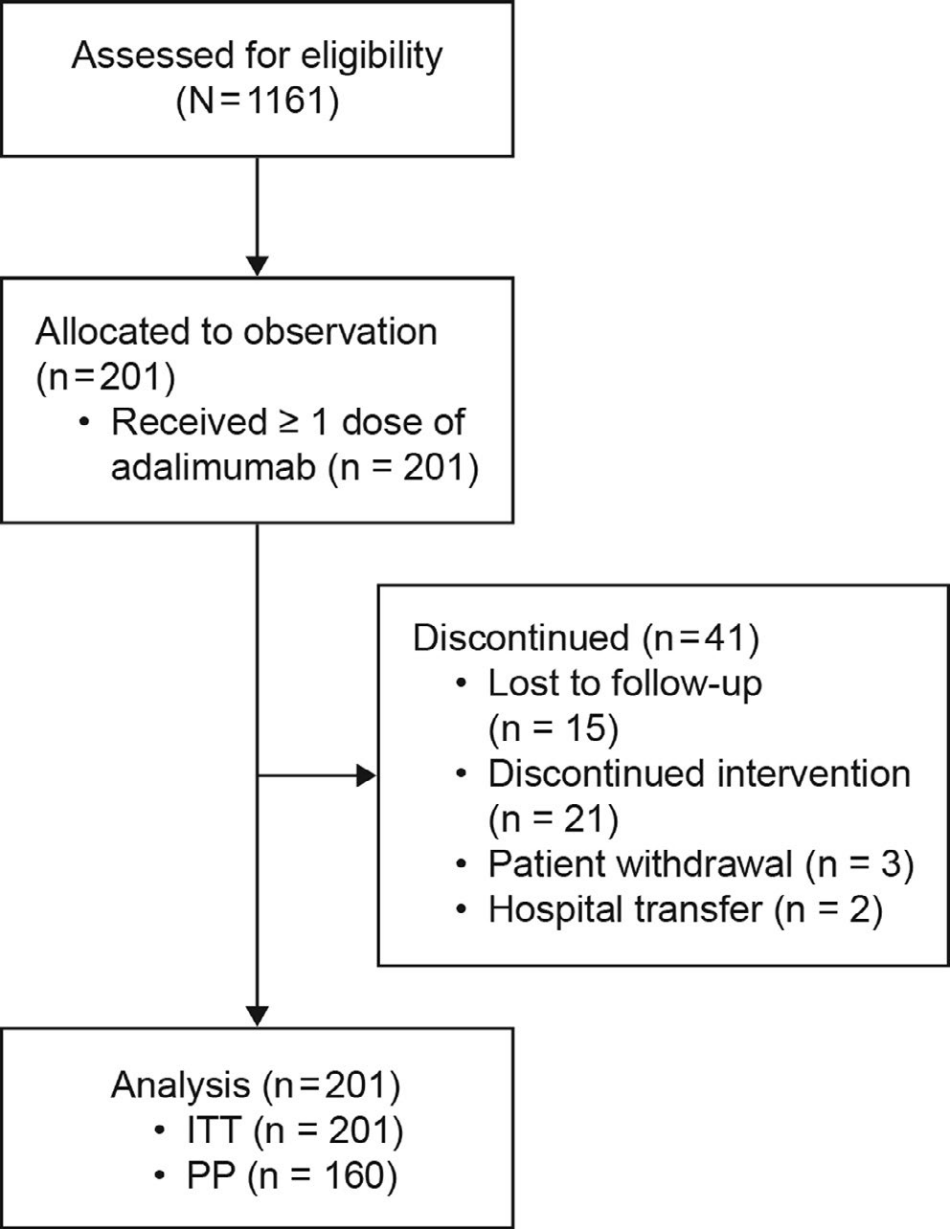
Patient flow diagram. ITT, intention‐to‐treat; PP, per protocol

The eventual enrolled population comprised 201 patients, all of whom received at least 1 dose of adalimumab and were included in the ITT population. The PP population consisted of 160 patients, as described in the patient flow diagram (Figure 1). Overall, 41 patients did not complete the study, of whom 15 were lost to follow‐up. Patient characteristics and demographics at baseline are summarized in Table 1. The mean (SD) age was 39.8 (12.3) years, and 80.6% (162) of patients were male. At baseline, 41.3% of patients presented with comorbidities, including 13 patients (6.5%) with uveitis, 11 (5.5%) with gastrointestinal disorders, and 5 (2.5%) with skin and subcutaneous tissue disorders. All patients had received prior therapy for AS, and 38 patients (18.9%) had received previous disease‐modifying antirheumatic drug treatment. Most patients (84.6%) had been treated with concomitant NSAIDs, and 35.8% had received concomitant corticosteroid treatment for AS.

**TABLE 1:** Patient baseline characteristics and demographics

	Patients	
	Enrolled (n = 201)	Total (N = 1161)
Age, y	39.8 (12.3)	40.5 (12.1)
Male	162 (80.6)	941 (81.1)
Concomitant NSAID	170 (84.6)	
Concomitant corticosteroids	72 (35.8)	
Prior TNFα inhibitor therapy	34 (17.0)	
Etanercept	17 (8.5)	
Infliximab	10 (5.0)	
Adalimumab	8 (4.0)	
Golimumab	4 (2.0)	
Comorbidities	83 (41.3)	
Eye disorders	17 (8.5)	
Uveitis	13 (6.5)	
Gastrointestinal disorders	11 (5.5)	
Crohn's disease	1 (0.5)	
Hypertension	24 (11.9)	
Diabetes mellitus	7 (3.5)	
Skin and subcutaneous tissue disorders	5 (2.5)	
Psoriasis	3 (1.5)	

Note

Data are n (%) or mean ± SD.

Abbreviations: NSAID, nonsteroidal anti‐inflammatory drug; SD, standard deviation; TNFα, tumor necrosis factor‐alpha.

### 3.2 Frequency of peripheral disease

From the original 1161 pool of patients with AS, 428 (36.9%) had peripheral disease features at baseline (Table 2). Of these, 15.3% presented with enthesitis, 26.4% with peripheral arthritis, and 2.4% with dactylitis. Within the enrolled ITT population (n = 201), 46.3% presented with enthesitis, 33.3% had peripheral arthritis, and 3.0% had dactylitis.

**TABLE 2:** Prevalence of peripheral disease at baseline

	Patients, n (%)	
	Enrolled (n = 201)	Total (N = 1161)
Presence of peripheral disease	‐	428 (36.9)
Peripheral involvement^a^		
Enthesitis	93 (46.3)	178 (15.3)
Peripheral arthritis	67 (33.3)	306 (26.4)
Dactylitis	6 (3.0)	28 (2.4)

^a^ Patients could present with > 1 peripheral disease feature.

### 3.3 Adalimumab significantly reduced AS disease activity

**FIGURE 2:**
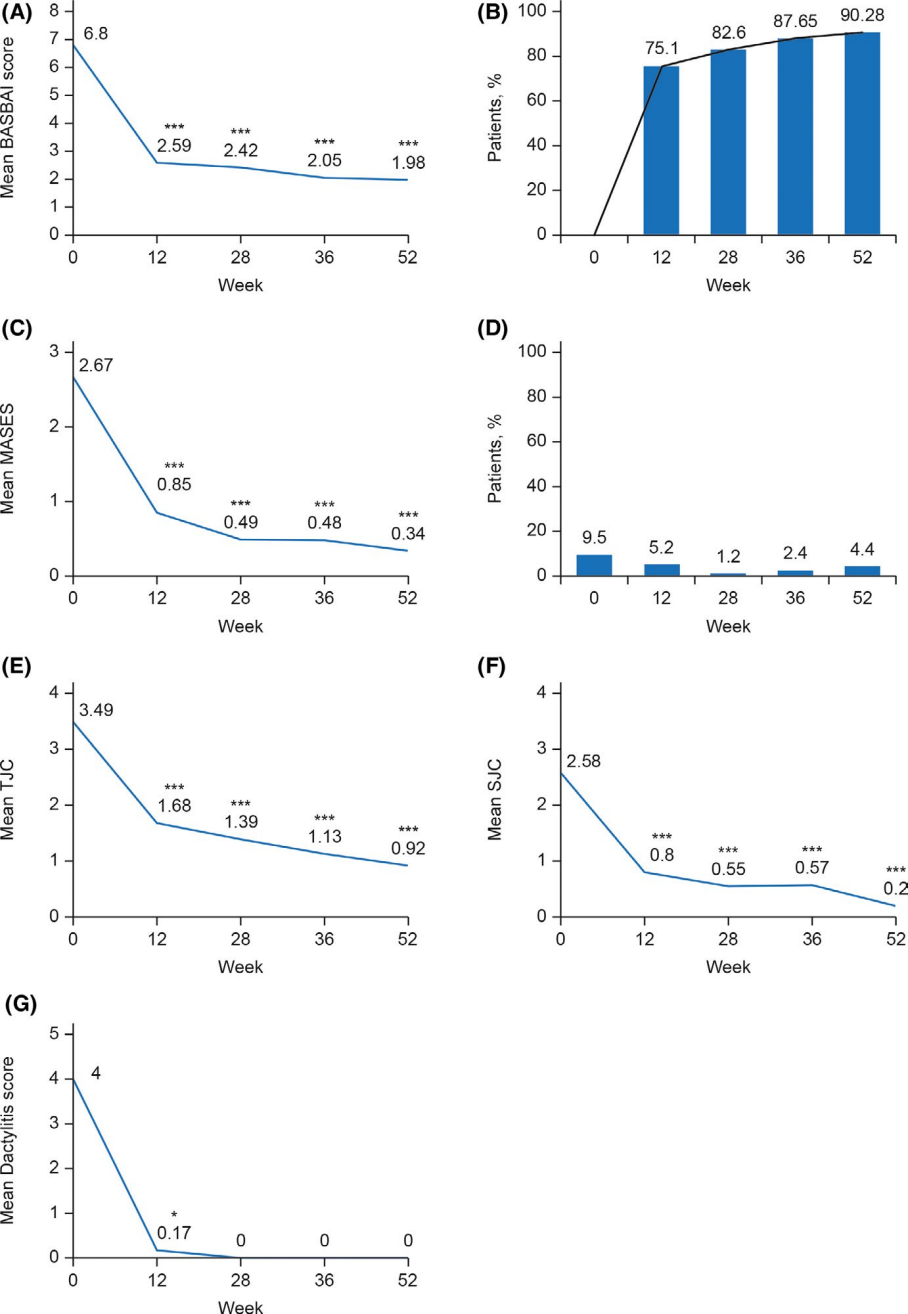
Assessment of ankylosing spondylitis (AS) and peripheral disease over 52 weeks. A, Mean Bath Ankylosing Spondylitis Disease Activity Index (BASDAI) score from baseline to 52 weeks. B, Patients with AS achieving 50% improvement in BASDAI score (BASDAI 50) over the treatment duration. C, Change in Maastricht Ankylosing Spondylitis Enthesitis Score (MASES) for patients with enthesitis at baseline. D, Prevalence of enthesitis of the plantar fascia across the study duration (%). E, Changes in tender joint count (TJC) and F, swollen joint count (SJC) in patients presenting with peripheral arthritis (≥1 swollen joint) from baseline. G, Change in dactylitis score for patients who presented with dactylitis at baseline. *P* values were calculated by paired *t* test or signed rank test (*P < .05; ***P < .0001) and represent the differences between baseline and values assessed at study's subsequent visits.

Responses to adalimumab treatment were assessed by improvement in BASDAI score from baseline. Mean (SD) BASDAI score for the ITT population (n = 201) was 6.80 (1.40) at baseline, which decreased to 1.98 (1.27) (*P* < .0001) by study end at week 52 (n = 144; Figure 2A and Table S2). Mean BASDAI score was shown to improve significantly between weeks 0 and 12, with a mean (SD) decrease of −4.24 (1.68). BASDAI score improved further over the full study duration (mean [SD] decrease, −4.82 [1.55]). Changes from baseline in the mean BASDAI score for the PP set (data not shown) were found to be similar as for the ITT population.

By week 52, most patients (90.3%) exhibited BASDAI 50 compared with scores at baseline, with 75.1% (142/189) of patients achieving BASDAI 50 by week 12 (Figure 2B).

We further studied in a *post hoc* analysis adalimumab efficacy in patient subgroups stratified according to the number of study drug administrations received. Patients were divided into 3 groups: ≤13 doses (n = 23), 14 to 25 doses (n = 20), and ≥26 (n = 158) doses. By week 12, more patients in the 14 to 25 (80.0%, 16/20) and ≥26 (77.3%, 119/154) dose groups achieved BASDAI 50, compared with patients who received ≤13 doses of adalimumab (46.7%, 7/15; *P* < .0440; Table S3 and Figure S1). Similarly, the group that received ≥26 doses of adalimumab had the highest proportion of patients who achieved BASDAI 50 at week 28 (83.3%, 115/138; *P* < .0402).

In an analysis of adalimumab efficacy in patient subgroups stratified by prior use of TNFα inhibitors, a higher proportion of TNFα inhibitor‐naïve patients (77.2%, 122/158) achieved BASDAI 50 at week 12, compared with those who had switched from other TNFα inhibitor therapies (64.5%, 20/31; Table S4). There was no statistically significant difference in adalimumab efficacy based on a prior use of TNFα inhibitors (Table S5).

### 3.4 Adalimumab efficacy in peripheral disease

Adalimumab was found to be effective in the treatment of peripheral disease in Korean AS patients over the duration of the study. Of 86 patients diagnosed with enthesitis at baseline (mean [SD] MASES of 2.67 [1.88]), adalimumab treatment resulted in a mean (SD) reduction in MASES of −2.50 (1.90) at week 52 (*P* < .0001; Figure 2C and Table S2). At study entry, 9.5% patients had enthesitis of the plantar fascia. By week 12, this had reduced to 5.2%, and by treatment end reduced to 4.4% of patients with this peripheral disease feature (Figure 2D).

Peripheral arthritis was diagnosed in 67 patients at study enrollment. At baseline, mean (SD) TJC was 3.49 (2.74), which significantly decreased to 0.92 (1.71) by week 52 (mean [SD] reduction of −2.60 [2.87]; *P* < .0001; Figure 2E and Table S2). Similarly, SJC decreased significantly over the study period (mean [SD] difference of −2.40 [2.30]; *P* < .0001; Figure 2F and Table S2). Adalimumab was curative for dactylitis in the 6 patients who presented with this feature at baseline (mean [SD] score of 4.00 [3.52]). By week 12, mean (SD) dactylitis score had decreased to 0.17 (0.41) (*P* < .0313). This effect was stable for the duration of the study (Figure 2G and Table S2).

### 3.5 Safety and tolerability of adalimumab

There were 22 AEs reported in 18 patients (9.0%) during the study (Table 3). Most were mild or moderate, and resolvable (86.4%). Serious adverse events (SAEs) were reported in 8 patients (4.0%, 11 events). Three SAEs were classed as severe and comprised chronic myelomonocytic leukemia in a patient with a positive hereditary cancer test, a lymphadenopathy, and a road traffic accident.

**TABLE 3:** Summary of AEs and SAEs

	Patients n (%)	AEs n
AE	18 (9.0)	22
Adverse drug reaction^a^	14 (7.0)	17
SAE	8 (4.0)	11
Serious adverse drug reaction^a^	5 (2.5)	7
AE leading to the discontinuation^b^	12 (6.0)	13

Abbreviations: AE, adverse event; SAE, serious adverse event.

^a^ Adverse drug reaction: AE the causality with adalimumab of which is probable, possible, probably not, or not assessable.

^b^ AE leading to discontinuation of adalimumab: an AE for which the action taken was transiently discontinued or permanently discontinued.

Seventeen (77.3%) AEs were classified as adverse drug reactions (ADRs; n = 14). Most common ADRs were skin and subcutaneous tissue disorders (23.5%), followed by infections and infestations (17.6%), general disorders, and injection site reactions (11.8%). ADRs led to interruption of study treatment in 4 patients (23.5% of ADRs), and discontinuation in 9 patients (52.9% of ADRs).

### 3.6 Adalimumab drug survival and patient discontinuation

**FIGURE 3:**
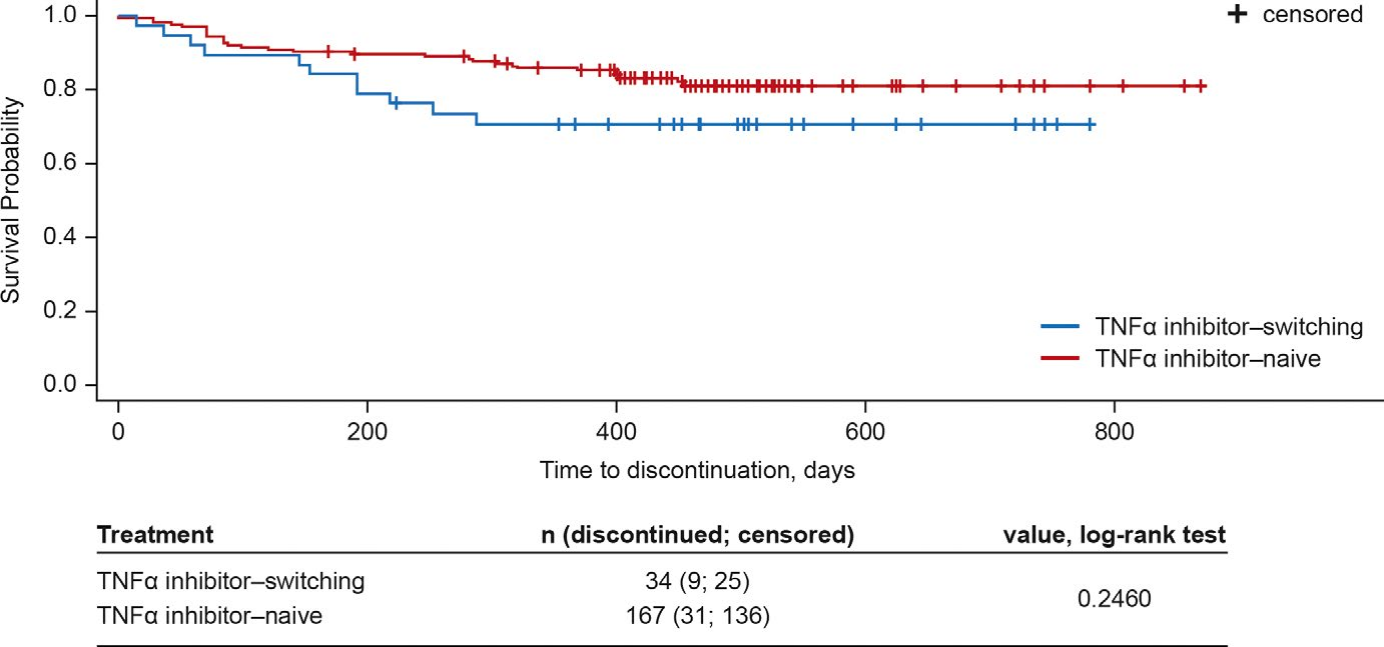
Kaplan–Meier survival probability for adalimumab adherence. Tick marks reflect patient adalimumab discontinuation, censored symbols denote a patient's last follow‐up date even though the patient continued the drug treatment. Change of Maastricht Ankylosing Spondylitis Enthesitis Score, dactylitis score, tender joint count, and swollen joint count was tested with the paired t test or signed rank test (*P < .05). TNFα, tumor necrosis factor‐alpha

For the ITT population, the median total administration frequency was 32 (range 1‐62) injections of adalimumab, and the median total dose administered was 1280 mg (range 40‐2480 mg). Adalimumab retention rate over the study period was assessed post hoc using Kaplan–Meier analysis, which showed that most patients persisted with therapy beyond the 52‐week study period. In an analysis stratified by prior use of TNFα inhibitors, drug survival was numerically superior in TNFα inhibitor‐naïve patients, but not significantly different for those who had switched from other TNFα inhibitor therapies (Figure 3).

## 4 DISCUSSION

Peripheral disease manifestations have been demonstrated as significant contributors to AS disease burden.^2^, ^5^ In this study, frequency of peripheral disease was assessed in an initial, larger patient population to provide a more representative proportion of Korean patients with AS. Of those 1161 patients, over one‐third (36.9%, n = 428) were shown to have peripheral disease features; peripheral arthritis was most common (26.4%, n = 306), followed by enthesitis (15.3%, n = 178). Only 2.4% (n = 28) presented with dactylitis.

Previous longitudinal and cross‐sectional studies have reported a variable prevalence of peripheral disease manifestations, with peripheral arthritis reported in 13%–37%,^15^, ^16^, ^17^ enthesitis in 7%–74%,^15^, ^16^, ^18^ and dactylitis in 4%–6%^16^, ^17^ of patients with AS. A meta‐analysis of these studies has shown comparable prevalence of peripheral arthritis (29.7%) and enthesitis (28.8%).^11^ While enthesitis is the primary lesion in AS, its prevalence in the current study was lower than that of arthritis. Data from the nationwide Korean College of Rheumatology BIOlogics (KOBIO) registry revealed similar results with enthesitis reported in 21% and peripheral arthritis in 35% of patients with AS.^14^ This difference could be explained by the variability in symptom duration and disease severity. Considering the broad patient population included in the current study, the prevalence of peripheral disease is in line with the frequencies reported in the pooled analysis of the global studies in AS.^11^

Patients enrolled in the study were those deemed eligible for adalimumab treatment; thus, the frequency of peripheral involvement was found to be higher in the ITT population (n = 201), with enthesitis diagnosed in 46.3% of patients, followed by peripheral arthritis in 33.3%. Two previous studies have examined prevalence of peripheral disease among AS patients in Korea. In line with our findings for the ITT population, enthesitis was observed in 42.7% and 53% of patients with AS, respectively.^3^, ^4^ However, in both studies, the frequency of peripheral arthritis was found to be higher (47.1% and 70%, respectively). These differences may reflect changes in the clinical management of AS over recent years, as well as variations in patient characteristics between the studies. In terms of other comorbidities, 6.5% of patients enrolled in the study had uveitis, lower than the prevalence of 29% previously reported by Koo et al in a single‐center study in Korean patients with AS.^19^ The study may represent patients with severe disease and a long disease duration, which may explain the higher prevalence of uveitis reported.^19^

In line with prior reports, adalimumab was effective for the treatment of AS in this population of Korean patients, with significant decreases in mean BASDAI score and clinical responses (BASDAI 50) determined in approximately 90% of patients by week 52.^7^ An additional post hoc subgroup analysis demonstrated that at weeks 12 and 28, significantly more patients achieved clinical improvement in BASDAI score (BASDAI 50) if they adhered to the recommended dosing schedule for adalimumab.

This study is the first to assess the efficacy of adalimumab on peripheral joint involvement (peripheral arthritis, enthesitis, or dactylitis) in Korean patients with AS. Adalimumab therapy was associated with substantial improvement in all evaluated peripheral disease features. Enthesitis was the most prevalent peripheral disease at baseline (46.3% of enrolled patients). As indicated by change in MASES, enthesitis was significantly improved with adalimumab, in particular during the initial 12 weeks of treatment. Significant improvements were also shown in patients with peripheral arthritis features, and dactylitis was completely resolved in affected patients by week 28. The efficacy of adalimumab on peripheral joint involvement in AS is in agreement with findings of a prior study in a European population, which reported BASDAI 50 for 55.7% of patients with enthesitis and peripheral arthritis at week 12.^13^ TNFα inhibitors are comparable in their efficacy with regard to peripheral disease. Data is lacking for other biologics such as interleukin‐17 (IL‐17) inhibitors for their efficacy with regard to peripheral disease in AS.^20^, ^21^ However, studies on patients with psoriatic arthritis showed beneficial effects of secukinumab (in particular with initial subcutaneous loading doses), with resolution of enthesitis observed in more than 50% of patients by week 16.^22^, ^23^ This may not be a class‐effect as similar results were not observed with other IL‐17 inhibitors such as ixekizumab.^24^

Adalimumab adherence resulted in improved outcomes for patients, with more patients achieving BASDAI 50 at weeks 12 and 28 if they received more than 13 doses of adalimumab treatment, compared with patients who received ≤13 doses. A prior study compared retention rates following TNFα inhibitor treatment (etanercept, infliximab, and adalimumab) in rheumatoid arthritis, psoriatic arthritis and AS, and found the cumulative survival to favor AS patients.^25^ The study reported a 28.6% withdrawal rate for patients with AS on adalimumab, which is higher than the 20.4% discontinuation rate observed in the present study. Withdrawal rates were found to be lower for AS patients treated with infliximab and etanercept, 19.5% and 24.6%, respectively; however, of the AS patients studied (n = 249), only 14 received adalimumab treatment.^25^ In another real‐world evidence study assessing the adherence and persistence of adalimumab across different indications, 78% of patients with AS (n = 203) showed a good adherence (medication possession ratio of ≥80%) to adalimumab treatment over a median follow‐up of 3.07 years.^26^ In addition, the median time to discontinuation of therapy (stopped treatment for ≥180 days) was longest for patients with AS (27 months).^26^ Taken together, the evidence shows a high adherence and persistence rates with adalimumab therapy in AS, reflecting high efficacy and satisfactory safety profile of this therapy in AS. Furthermore, the efficacy of adalimumab treatment based on BASDAI 50 responses was consistent in TNFα inhibitor‐naïve patients, as well as in those who had switched from other TNFα inhibitor therapies. Previous studies in Korean patients with AS have shown lower rates for switching to other TNFα inhibitors in patients who had received adalimumab as first‐line therapy. In addition, most patients who switched from other TNFα inhibitors went on to receive adalimumab as second‐line therapy.^27^, ^28^

Adalimumab treatment was well tolerated in this population of Korean patients with AS, with no new safety signals observed. Most common ADRs were skin and subcutaneous tissue disorders (23.5%), which were mostly resolvable (75%). The incidence of SAEs was low (4.0%), and the proportions of patients who developed study drug‐related infections or injection site reactions were of a similar magnitude to a previous study of adalimumab in AS.^8^

The strengths of this study are its noninterventional nature, and the broad patient population gathered from multiple centers across Korea. Our findings add to the limited current evidence surrounding prevalence of peripheral disease features among Korean patients with AS. This is also the first study to examine the efficacy of adalimumab on peripheral disease in this ethnic population. However, there were study limitations, which include potential variations in reporting across the 13 centers. It is also of note that the post hoc analyses were not powered for statistical significance. In order to confirm the findings observed, an appropriately powered study in a larger patient population would be required.

In summary, in this noninterventional, observational study of Korean patients with AS, enthesitis and peripheral arthritis were the most prevalent peripheral disease features, both occurring in more than one‐third of patients. Adalimumab treatment was shown to be effective in reducing spinal symptoms and peripheral disease in these subjects.

## 5 DATA ACCESSIBILITY STATEMENT

The authors declare that all data supporting the findings of this study are available within the article and its supplementary information files.

## CONFLICTS OF INTEREST

Yusun Lee is an employee of AbbVie Ltd and may own AbbVie stock or options. All other authors do not have any conflict of interest to declare.

## AUTHOR CONTRIBUTIONS

Conceptualization: Kim TH. Data curation: Lee SH, Kim TH. Formal analysis: Lee SH. Funding acquisition: Kim TH. Investigation: Lee SH, Park W, Lee SW, Kim HA, Choe JY, Lee SH, Lee SS, Park SH, Park MC, Sheen DH, Lee HS, Lee YA, Kim TH. Project administration: Park W, Lee SW, Kim HA, Choe JY, Lee SH, Lee SS, Park SH, Park MC, Sheen DH, Lee HS, Lee YA, Lee YS, Kim TH. Supervision: Kim TH. All authors participated in interpreting the data, in writing and critically reviewing the manuscript, and approved the final version.

## Supporting information

Supinfo
