# Supplementary material for: Frequency of peripheral diseases in Korean patients with ankylosing spondylitis and the effectiveness of adalimumab
Source: Int J Rheum Dis. 2020 Jul 29;23(9):1175–83. doi: 10.1111/1756-185X.13917 (PMC8246777; doi:10.1111/1756-185X.13917)

**Frequency of peripheral diseases in Korean patients with ankylosing spondylitis and the effectiveness of adalimumab**

# SUPPLEMENTARY INFORMATION

# Supplementary Table 1. Study sites

| Study institutions |
| --- |
| Chonnam National University Hospital |
| Daegu Catholic University Medical Center |
| Dong-A University Hospital |
| Eulji University Hospital |
| Gangnam Severance Hospital |
| Hallym University Sacred Heart Hospital |
| Hanyang University Guri Hospital |
| Hanyang University Seoul Hospital |
| Inha University Hospital |
| Konkuk University Medical Center |
| Kyung Hee University Hospital |
| Kyung Hee University Hospital at Gangdong  Seoul St. Mary's Hospital, The Catholic University of Korea |

# Supplementary Table 2. Assessment of AS and peripheral disease over 52 weeks.

|  | **Week** | | | | |
| --- | --- | --- | --- | --- | --- |
|  | **0** | **12** | **28** | **36** | **52** |
| BASDAI score | 6.80 (1.40) | 2.59 (1.66)*** | 2.42 (1.57)*** | 2.05 (1.34)*** | 1.98 (1.27)*** |
| MASES | 2.67 (1.88) | 0.85 (1.86)*** | 0.49 (0.90)*** | 0.48 (1.44)*** | 0.34 (1.02)*** |
| Dactylitis score | 4.00 (3.52) | 0.17 (0.41)* | 0 | 0 | 0 |
| TJC | 3.49 (2.74) | 1.68 (2.95)*** | 1.39 (2.95)*** | 1.13 (2.50)*** | 0.92 (1.71)*** |
| SJC | 2.58 (2.11) | 0.80 (1.17) | 0.55 (2.14) | 0.57 (1.97) | 0.20 (0.76) |

Data are mean ± SD. P-values were calculated by paired t-test or signed rank test (**P* <0.05; ****P* <0.0001) and represent the differences between baseline and values assessed at study’s subsequent visits.

AS, ankylosing spondylitis; BASDAI, Bath Ankylosing Spondylitis Disease Activity Index; MASES, Maastricht Ankylosing Spondylitis Enthesitis Score; SD, standard deviation; SJC, swollen joint count; TJC, tender joint count (TJC).

# Supplementary Table 3. *Post hoc* analysis of the proportion of patients achieving 50% improvement in the Bath Ankylosing Spondylitis Disease Activity Index score (BASDAI 50) according to doses of adalimumab received over 52 weeks

| **BASDAI 50** | **Patient subgroup** | | | **P-value, Fisher exact test** |
| --- | --- | --- | --- | --- |
|  | **≤13 doses** | **14–25 doses** | **≥26 doses** |  |
|  | **(n=23)** | **(n=20)** | **(n=158)** |  |
| Week 12 |  |  |  |  |
| n^†^ | 15 | 20 | 154 | 0.0440 |
| % | 46.7 | 80.0 | 77.3 |  |
| Week 28 |  |  |  |  |
| n^†^ | 2 | 15 | 138 | 0.0402 |
| % | 0.0 | 86.7 | 83.3 |  |
| Week 36 |  |  |  |  |
| n^†^ | 0 | 11 | 151 | 0.1390 |
| % | 0.0 | 72.7 | 88.7 |  |
| Week 52 |  |  |  |  |
| n^†^ | 0 | 4 | 140 | 1.0000 |
| % | 0.0 | 100.0 | 90.0 |  |

^†^n represents the population assessed for each subgroup.

BASDAI 50, 50% improvement in Bath Ankylosing Spondylitis Disease Activity Index score

# Supplementary Table 4. *Post hoc* analysis of the proportion of patients achieving 50% improvement in the Bath Ankylosing Spondylitis Disease Activity Index score (BASDAI 50) according to prior TNFα inhibitor therapy over 52 weeks

| **BASDAI 50** | **Patient subgroup** | | **P-value, Fisher exact test** |
| --- | --- | --- | --- |
|  | **TNFα inhibitor–switching** | **TNFα inhibitor–naive** |  |
|  | **(n=34)** | **(n=167)** |  |
| Week 12 |  |  |  |
| n^†^ | 31 | 158 | 0.1348^‡^ |
| % | 64.5 | 77.2 |  |
| Week 28 |  |  |  |
| n^†^ | 25 | 130 | 1.000 |
| % | 84.0 | 82.3 |  |
| Week 36 |  |  |  |
| n^†^ | 25 | 137 | 0.5169 |
| % | 84.0 | 88.3 |  |
| Week 52 |  |  |  |
| n^†^ | 22 | 122 | 1.000 |
| % | 90.9 | 90.2 |  |

BASDAI 50, 50% improvement in Bath Ankylosing Spondylitis Disease Activity Index score; TNFα, tumor necrosis factor–alpha.

^†^n represents the population assessed for each subgroup.

^‡^Chi-square test, P-value.

# Supplementary Table 5. *Post hoc* analysis for the intra- and inter-group study difference in efficacy of endpoints for patients with and without exposure to prior TNFα inhibitor therapy over 52 weeks

|  | **TNFα inhibitor–switching, n=34** | | |  | **TNFα inhibitor–naive, n=167** | | | |  | |  |  |
| --- | --- | --- | --- | --- | --- | --- | --- | --- | --- | --- | --- | --- |
|  |  | **Difference from baseline** | |  | |  | **Difference from baseline** | | | **P-value^‡^** | |  |
|  | **n** | **Mean ± SD** | **P-value^†^** |  | | **n** | **Mean ± SD** | **P-value^†^** | |  |  |  |
| BASDAI score | |  |  |  |  | |  |  |  | |  | |
| Week 12 | | 31 | −3.91±1.89 | <0.0001 |  | | 158 | −4.31±1.64 | <0.0001 | | 0.2292 | |
| Week 26 | 25 | −4.29±1.32 | <0.0001 |  | | 130 | −4.48±1.82 | <0.0001 | | 0.6118 | |  |
| Week 36 | 25 | −4.52±1.38 | <0.0001 |  | | 137 | −4.80±1.51 | <0.0001^§^ | | 0.6850^‖^ | |  |
| Week 52 | 22 | −4.61±1.00 | <0.0001 |  | | 122 | −4.86±1.63 | <0.0001 | | 0.3432 | |  |
| MASES |  |  |  |  | |  |  |  | |  | |  |
| Week 12 | 11 | −1.18±0.60 | 0.0020^§^ |  | | 73 | −1.95±2.66 | <0.0001^§^ | | 0.1091^‖^ | |  |
| Week 26 | 11 | −1.27±0.79 | 0.0039^§^ |  | | 61 | −2.49±2.13 | <0.0001^§^ | | 0.0537^‖^ | |  |
| Week 36 | 10 | −1.50±0.85 | 0.0003 |  | | 61 | −2.44±1.86 | <0.0001^§^ | | 0.1440^‖^ | |  |
| Week 52 | 10 | −1.70±0.82 | 0.0020^§^ |  | | 58 | −2.64±2.01 | <0.0001^§^ | | 0.1727^‖^ | |  |
| Dactylitis score |  |  |  |  | |  |  |  | |  | |  |
| Week 12 | 1 | −2.00 | NA |  | | 5 | −4.20±3.96 | 0.0768 | | 0.7664^‖^ | |  |
| Week 26 | 1 | −2.00 | NA |  | | 4 | −5.00±4.08 | 0.0917 | | 0.4682^‖^ | |  |
| Week 36 | 0 | NA | NA |  | | 4 | −5.00±4.08 | 0.0917 | | NA | |  |
| Week 52 | 0 | NA | NA |  | | 4 | −5.00±4.08 | 0.0917 | | NA | |  |
| TJC |  |  |  |  | |  |  |  | |  | |  |
| Week 12 | 9 | −3.56±3.40 | 0.0039^§^ |  | | 57 | −1.54±3.38 | <0.0001^§^ | | 0.0941^‖^ | |  |
| Week 26 | 9 | −4.33±3.57 | 0.0066 |  | | 47 | −1.62±2.75 | <0.0001^§^ | | 0.0377^‖^ | |  |
| Week 36 | 8 | −4.50±4.04 | 0.0161 |  | | 45 | −2.00±2.84 | <0.0001^§^ | | 0.1564^‖^ | |  |
| Week 52 | 8 | −4.50±4,63 | 0.0285 |  | | 42 | −2.24±2.31 | <0.0001^§^ | | 0.3345^‖^ | |  |
| SJC |  |  |  |  | |  |  |  | |  | |  |
| Week 12 | 9 | −2.22±2.49 | 0.0078^§^ |  | | 57 | −1.74±2.23 | <0.0001^§^ | | 0.7653^‖^ | |  |
| Week 26 | 9 | −2.22±2.49 | 0.0078^§^ |  | | 47 | −1.94±2.86 | <0.0001^§^ | | 0.2710^‖^ | |  |
| Week 36 | 8 | −2.13±2.85 | 0.0729^§^ |  | | 45 | −2.02±2.89 | <0.0001^§^ | | 0.3870^‖^ | |  |
| Week 52 | 8 | −2.38±2.62 | 0.0156^§^ |  | | 42 | −2.40±2.27 | <0.0001^§^ | | 0.3682^‖^ | |  |

BASDAI, Bath Ankylosing Spondylitis Disease Activity Index; MASES, Maastricht Ankylosing Spondylitis Enthesitis Score; NA, not applicable; SD, standard deviation; SJC, swollen joint count; TJC, tender joint count; TNFα, tumor necrosis factor–alpha.

^†^P-value measured intragroup; difference in paired *t* test (or ^§^signed rank test)

^‡^P-value measured intergroup; difference in *t* test (or ^‖^rank sum test)

# Supplementary Figure1. Patients with 50% improvement in the Bath Ankylosing Spondylitis Disease Activity Index score (BASDAI 50). Patients were divided into subgroups based on the number of adalimumab doses: ≤13, 14–25, and ≥26 doses. Outcomes for BASDAI 50 for weeks 12 and 28 are displayed.


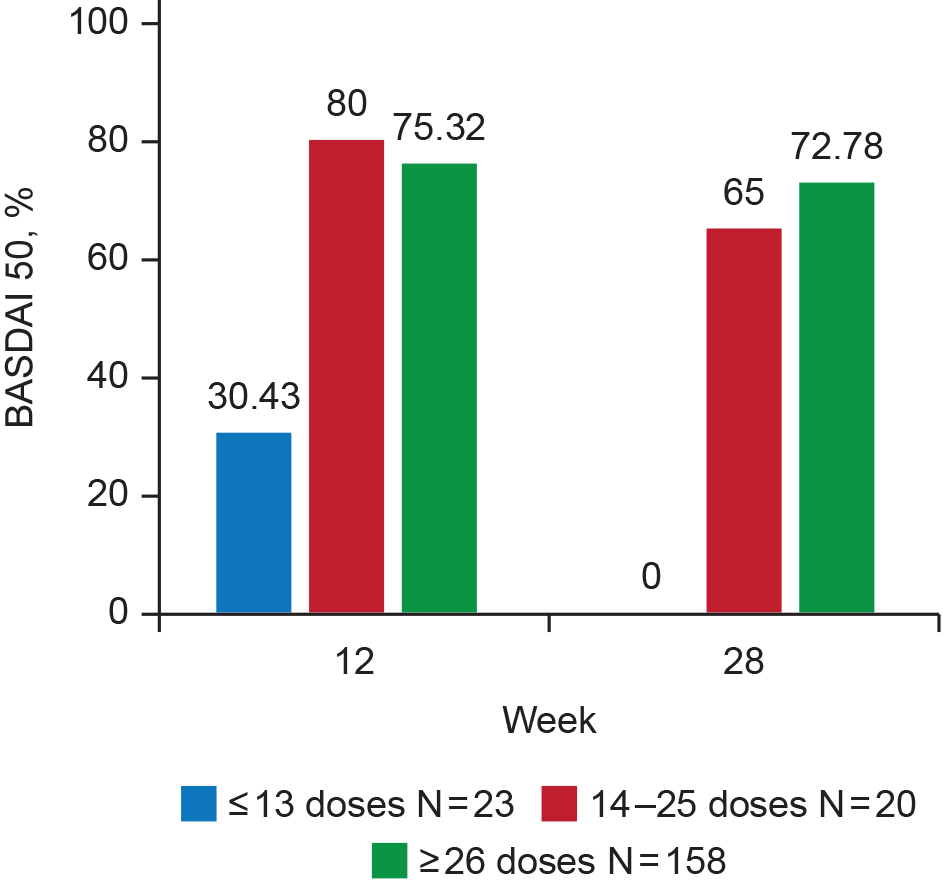

Supplement: Supplementary file 1 — Supinfo [file APL-23-1175-s001.docx]
